# Do the successful revision surgery for humeral nonunion solve all the effects on health‐related quality of life? A retrospective cohort study

**DOI:** 10.1186/s12891-021-04283-9

**Published:** 2021-05-05

**Authors:** Zhimeng Wang, Yao Lu, Liang Sun, Leilei Song, Teng Ma, Qiang Wang, Kun Zhang, Zhong Li

**Affiliations:** 1 Department of Orthopaedics and Trauma, Hong Hui Hospital, Xi’an Jiaotong University College of Medicine, No. 555, East Youyi Road, Shaanxi 710000 Xi’an, China; 2 Qinghai University, 810000 Xi’ning, Qinghai China

**Keywords:** Humerus, Nonunion, Quality of life, Revision surgery, SF-12

## Abstract

**Background:**

The aim of this study is to evaluate the effects of successful revision operation on health quality of life(QoL) and functional outcome in humeral nonunion patients.

**Methods:**

This retrospective study included 62 patients with humeral nonunion from Northwest China, who were admitted to the Department of Trauma Surgery, Honghui Hospital between March 2013 and September 2019. The following data were retrospectively evaluated: demographic data, clinical data, imaging findings, and treatment methods. The QoL assessment indicators for humeral nonunion patients included the SF-12 mental component summary (MCS) and physical component summary (PCS),brief pain inventory-severity(BPI-S) and brief pain inventory-interference (BPI-I). The mayo elbow performance score (MEPS) was used to assess the elbow function of the patients.

**Results:**

Successful revision surgery significantly improved the patient's PCS, MCS, BPI-S and BPI-I scores (*p*<0.001). According to the MEPS criteria, the excellent and good rates were 95.16% in this study. The impact of humeral nonunion on mental health was comparable with the reported impact of stroke and type II diabetes (*p*>0.05).The impact of post-op on physical health was comparable with the reported impact of COPD, silicosis, hypertension, barrentt’s esophagus and lower urinary tract symptoms(*p*>0.05).

**Conclusion:**

Humeral nonunion is a devastating chronic medical condition that negatively affects both physical and mental health as well as quality of life. Although the effects of pain in the body can be completely relieved by treatment, the entire medical process may cause everlasting psychological trauma to the patient.

## Background

Humeral fractures are common, and both conservative treatment and surgery can achieve good prognosis [1]. The humeral nonunion rate was up to 1 %~13 % among all fracture complications [2, 3]. Nonunion not only causes serious functional and financial burdens but also poses several problems for patients’ mental health and social stability [4–6]. However, the treatment of humeral nonunion is more often challenging, requiring one or more surgical interventions, which have high complication rates such as radial nerve palsy or joint stiffness [7].

Due to the unique problems of humeral nonunion, several treatment options are detailed in the literature [7–9]. The short and midterm surgical outcomes have been evaluated in most cases in terms of bone union, range of motion(ROM), infection rate and loss of strength. Nowadays, in China’s medical environment, surgical results and interests are often given first priority, ignoring the patient’s long-term pain and psychological trauma. Compared with other nonunion complications, pain has an important impact on the health status and quality of life(QoL) of patients, with chronic orthopedic injuries such as nonunion affecting psychosocial regulation and daily functions [5, 10].

There are few reports on the final follow-up results of QoL and functional outcomes of patients with humeral nonunion after repair operation. The purpose of this study was to evaluate the health-related QoL and functional outcomes in humeral nonunion patients.

## Patients and methods

### Patients

**Fig. 1:**
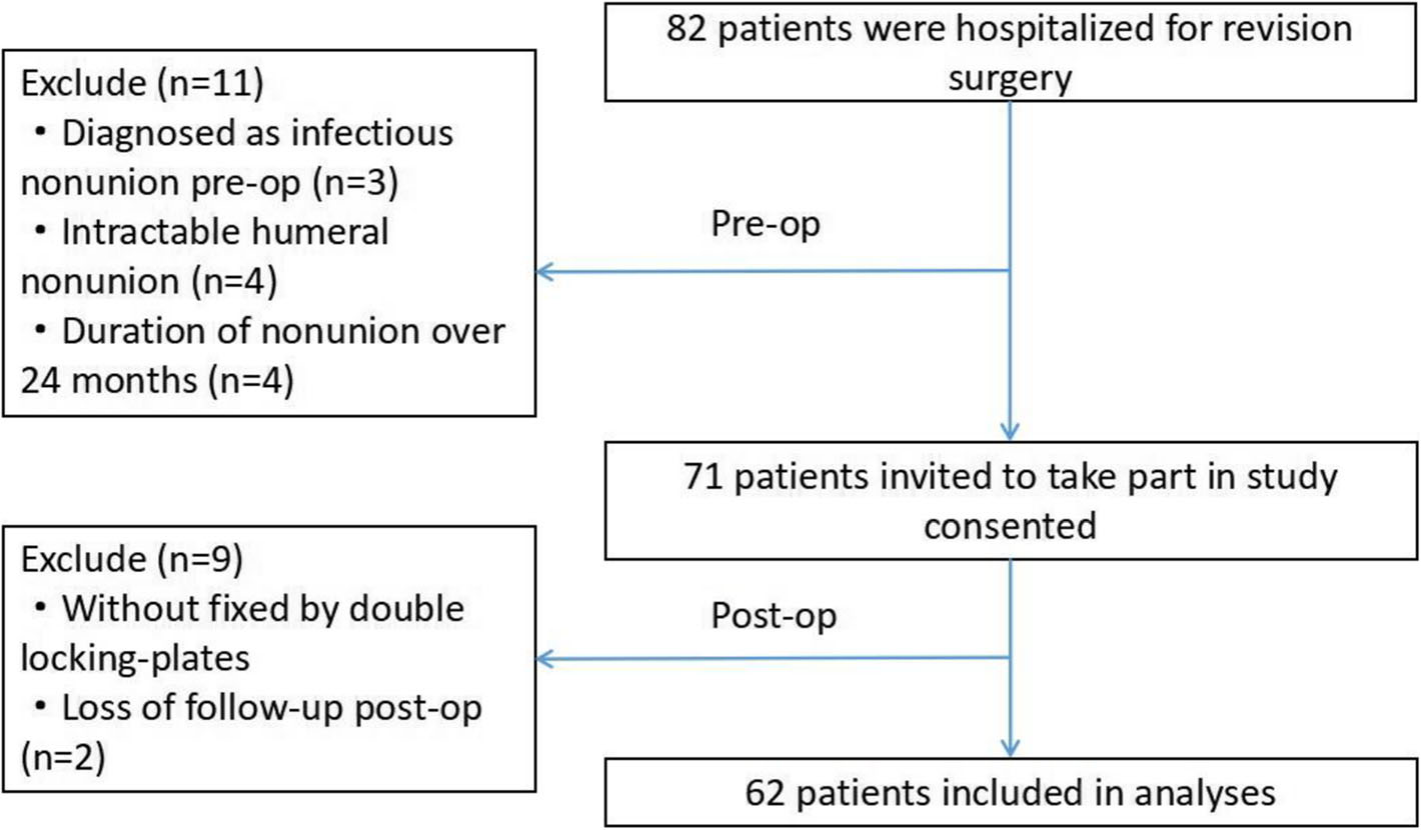
Flow chart of patient selection

A total of 62 patients who underwent revision surgery for humeral nonunion between March 2013 and September 2019 at the Department of Trauma Surgery, Hong Hui Hospital were included in this retrospective cohort study (Fig. 1).

### Study site

In China, hospitals are categorized in accordance with the medical level into three grades (third-grade is the best), and each grade is divided into three classes from good to bad: A, B, C. As a tertiary-A trauma center, Hong Hui Hospital is a designated trauma referral center in Northwest China.

### Inclusion criteria of humeral nonunion

(i)Patients with humeral nonunion from Northwest China. (ii)Older than 18 years and with certain cognitive abilities. (iii) We defined ‘‘nonunion’’ according to the US FDA (food and drug administration,FDA) criteria, where a fracture that is at least 9 months old has not shown any signs of healing for three consecutive monthsis [11].(iv) Patients who agreed to participate in this study and had complete follow-up data. (v) “Fresh nonunion” meant that patients had not undergone prior revision surgery.

### Exclusion criteria of humeral nonunion

(i)The original fracture type was pathological. (ii) Patients with physical conditions who could not tolerate surgery. (iii) Patients who had recently used glucocorticoids and immunosuppressive agents. (iv) Died halfway or quit halfway during the study.

### Fracture healing criteria

No local pain after holding heavy objects, no tenderness in the fracture space, imaging showed that at least three sides of the humerus had continuous osteophytes, no internal fixation failure [12–14].

### Ethics statement

This study was approved by the Ethics Committee of Honghui Hospital (No. 201,606,008) and conducted in accordance with the Declaration of Helsinki. All participating patients provided informed consent by signing a written informed consent form.

### Research methods

All patients with humeral nonunion who were treated with double-locking plates with channel bone grafting technology at our institution over one year period; the treatment had been completed and was retrospectively evaluated. The research team included three post-graduates, two attending physicians, and one chief physician, who were responsible for data collection, follow-up and revision surgery treatment. Thorough cleaning of fracture ends, satisfactory reduction, firm internal fixation and sufficient autogenous bone grafting was the treatment principle for humeral nonunion [15]. Follow-up methods included e-mail, telephone and outpatient visits, and the follow-up information was collected every three months.

The following patients’ characteristics were recorded: age, gender, smoking, alcohol abuse, body mass index (BMI), etiology, and comorbidities. The following clinical symptoms and performance were recorded: fracture type, primary therapeutic scheme, primary nonunion time, pathological type of nonunion, and autogenous bone grafting. The mayo elbow performance score (MEPS) was used to assess the elbow function of the patient in terms of range of activity, stability, pain and daily living ability [16]. Health-related QoL was evaluated using the short form-12 health survey (SF-12),this is because the general education level in Northwest China is low and cognitive level is not uniform, so the SF-12 scale has the advantages of easier understanding and acceptance, which is more suitable for patients with humeral nonunion [17]. The SF-12 scale has 12 entries and evaluates eight dimensions of health-related QoL: general health(GH), physiological function(PF),role physiological(RP),body pain(BP), vitality(VT), social function(SF),role emotional(RE) and mental health(MH). The physical component summary (PCS) consists of GH, PF, RP, and BP. The mental component summary (MCS) consists of SF, RE, MH and VT. The brief pain inventory(BPI) is a widely used basic questionnaire assessing pain interference, which includes 11 entries and two dimensions (BPI-S and BPI-I) [18].

In addition, we compared the SF-12 scores between Chinese humeral nonunion patients and a series of acute and chronic diseases [19–35], which allowed the doctors and patients to easily understand the extent to which humeral nonunion affected QoL.

### Statistical methods

Continuous variables are expressed as Mean ± SD and categorical variables as percentages (%). For parametric variables, Student’s t-test was used to compare between the groups. Unpaired T test was used to compare SF-12 scores of humeral nonunion patients with chronic disease patients. Significance was set at *p* < 0.05. All statistical analysis was performed using GraphPad Prism 8.0.

## Results

(i) Between March 2013 and September 2019, a total of 62 patients with humeral nonunion underwent revision surgery at our The patients were aged 20–73 years (mean 42.39 ± 4.17 years). There were 43 males and 19 females; of which 15 were smokers and seven were alcohol The average BMI was 25.71 ± 2.18 kg/m^2^. There were 36 cases of handness and 26 cases of non-handness; physical labor was high-risk group (37 cases, 59.68 %). Fall was the commonest cause of injury (43 cases, 69.4 %); hypertension was a common complication (11 cases, 17.7 %); closed fracture was the commonest fracture type (55 cases, 88.7 %). There were two cases of proximal nonunion, 51 cases of shaft nonunion, and seven cases of distal humerus. The average time from initial injury to nonunion was 7.39 ± 1.50 months (range, 6–16 months). There were 19 cases of atrophy, 14 cases of malnutrition, and 29 cases of hypertrophy. Local pain was the major complaint of most patients (39 cases, 62.90 %) (Tables 1 and 2).
(ii) Change of SF-12 and BPI scores: At the final follow-up, the SF-12 PCS, SF-12 MCS, BPI-S and BPI-I scores were 43.6 ± 8.1, 34.7 ± 4.4, 4.4 ± 2.1, and 4.1 ± 1.9, respectively, which were significantly improved as compared to preoperative scores (*p* < 0.001,Table 3). Although the final MCS score had also improved, the magnitude of the change was minimal.

**Table 1:** Patients’ characteristics

Patients’ characteristics		value(%)	
Age(mean ± SD, y)		42.29 ± 4.17	
Male/female		43/19	
BMI (mean ± SD, kg/m^2^)		25.71 ± 2.18	
Smoking			
yes		15(24.19 %)	
no		47(75.81 %)	
Alcohol abuse			
yes		7(11.29 %)	
no		55(88.71 %)	
Handness Inventory			
handness		36(58.06 %)	
non-handness		26(41.94 %)	
Occupation			
physical labor		37(59.68 %)	
mental worker		19(30.65 %)	
retirees		6(9.67 %)	
Etiology			
low fall		24(38.71 %)	
high fall		19(30.65 %)	
MCVs		8(12.90 %)	
fall objects		7(11.29 %)	
sport		4(6.45 %)	
Comorbidities			
hypertension		11(17.74 %)	
diabetes		8(12.90 %)	
heart disease		4(6.45 %)	
others^a^		4(6.45 %)	

Other^a^ included prostatitis, osteoporosis, hepatitis. Abbreviations: *BMI* body mass index, *MVCs* motor vehicle collisions

**Table 2:** Patients’ clinical data

Patients’ clinical data	value(%)
Fracture type	
closed	55(88.71 %)
open	7(11.29 %)
Fracture site	
proximal	2(3.23 %)
shaft	51(82.26 %)
distal	7(11.29 %)
Primary therapeutic scheme	
plaster fixation	4(6.45 %)
plate fixation	49(79.03 %)
intramedullary nail	3(4.84 %)
external fixation	6(9.68 %)
Duration of nonunion (mean ± SD, month)	7.39 ± 1.50
Pathological type of nonunion	
atrophic	19(30.65 %)
malnutrition	14 (22.58 %)
hypertrophic	29(46.77 %)
Chief complaint	
pain	39(62.90 %)
adynamia	20(32.26 %)
malformation pseudarthrosis	3(4.84 %)

**Table 3:** Comparison of SF-12 PCS, SF-12 MCS, BPI-S and BPI-I scores between pre-op and post-op in humeral nonunion patients

Variable	Pre-op	Post-op	*t value*	*p value*
SF-12 PCS (score, mean ± sd)	24.3 ± 5.2	43.6 ± 8.1	15.79	< 0.001
SF-12 MCS (score, mean ± sd)	26.3 ± 3.8	34.7 ± 4.4	11.38	< 0.001
BPI-S (score, mean ± sd)	6.5 ± 1.9	4.4 ± 2.1	5.839	< 0.001
BPI-I (score, mean ± sd)	6.7 ± 2.5	4.1 ± 1.9	6.520	< 0.001

“Post-op” means that the fracture ends were healed smoothly

(iii)Functional outcomes: The follow-up time of the 62 patients was 13–37 months, with an average of 18.3 ± 3.6 months. Finally, all patients with humeral nonunion successfully completed bone healing. The bone healing time was 6–13 months, with an average of 6.39 ± 1.5 months. The average elbow motion, forearm pronation and supination were 117.33 ± 13.9°, 83.57 ± 4.11°, and 77.21 ± 6.72°, respectively. According to the MEPS standard, 48 cases were graded as excellent, 11 cases as good, and three cases as fair. Excellent and good rates were 95.16 %.

(iv)Influence of Humeral Nonunion on Physical Health.

**Fig. 2:**
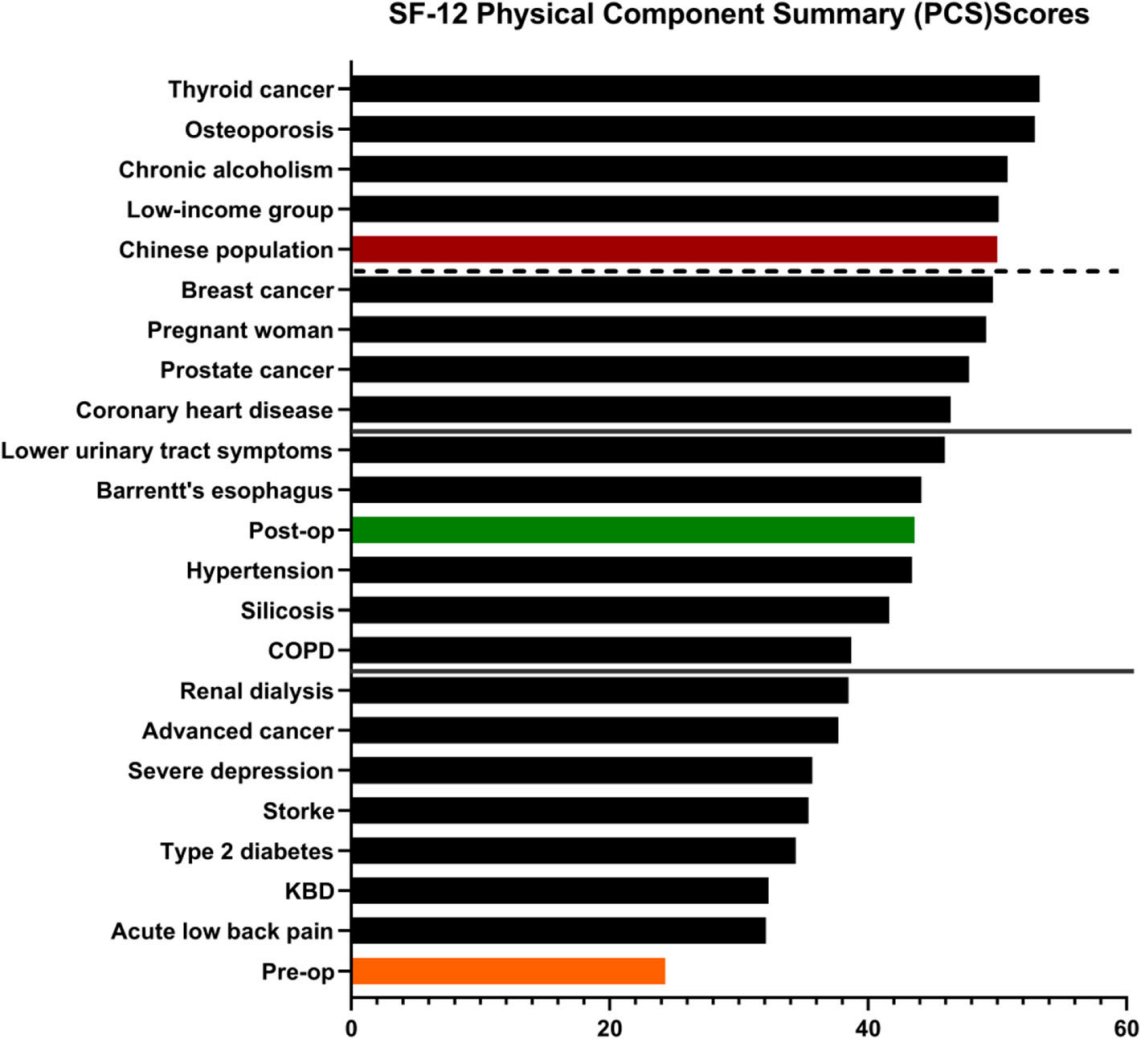
Mean SF-12 PCS scores according to diagnosis. Abbreviations: KBD, Kashin-Beck disease; COPD, Chronic Obstructive Pulmonary Disease. “Post-op” means that the fracture ends were healed smoothly. The medical conditions below the dashed line were associated with significantly (*p* < 0.05) better physical health compared with pro-op. Compared with post-op PCS, the medical condition above the upper solid line or below the lower solid line were associated with significantly (*p* <0.05) better or worse physical health, respectively

The preoperative SF-12 PCS score of humeral nonunion is the lowest of all included in the literature, and the postoperative score is at an intermediate level (Fig. 2). The PCS scores of diseases below the dashed line were significantly better than pro-op, and the differences were all statistically significant (*p* < 0.05).The mean impact of post-op on physical health was comparable with the reported impact of COPD, silicosis, hypertension, barrentt’s esophagus and lower urinary tract symptoms. The post-op impact was significantly worse than that of thyroid cancer (*p* < 0.001), osteoporosis (*p* < 0.001), chronic alcoholism (*p* < 0.001), low-income group (*p* < 0.001),breast cancer (*p* < 0.001), pregnant woman (*p* < 0.001), prostate cancer(*p* = 0.005),as well as coronary heart disease (*p* = 0.0361).Moreover, the mean pro-op and post-op PCS score was significantly below the theoretical Chinese population standard level.

(v) Influence of Humeral Nonunion on Mental Health.

**Fig. 3:**
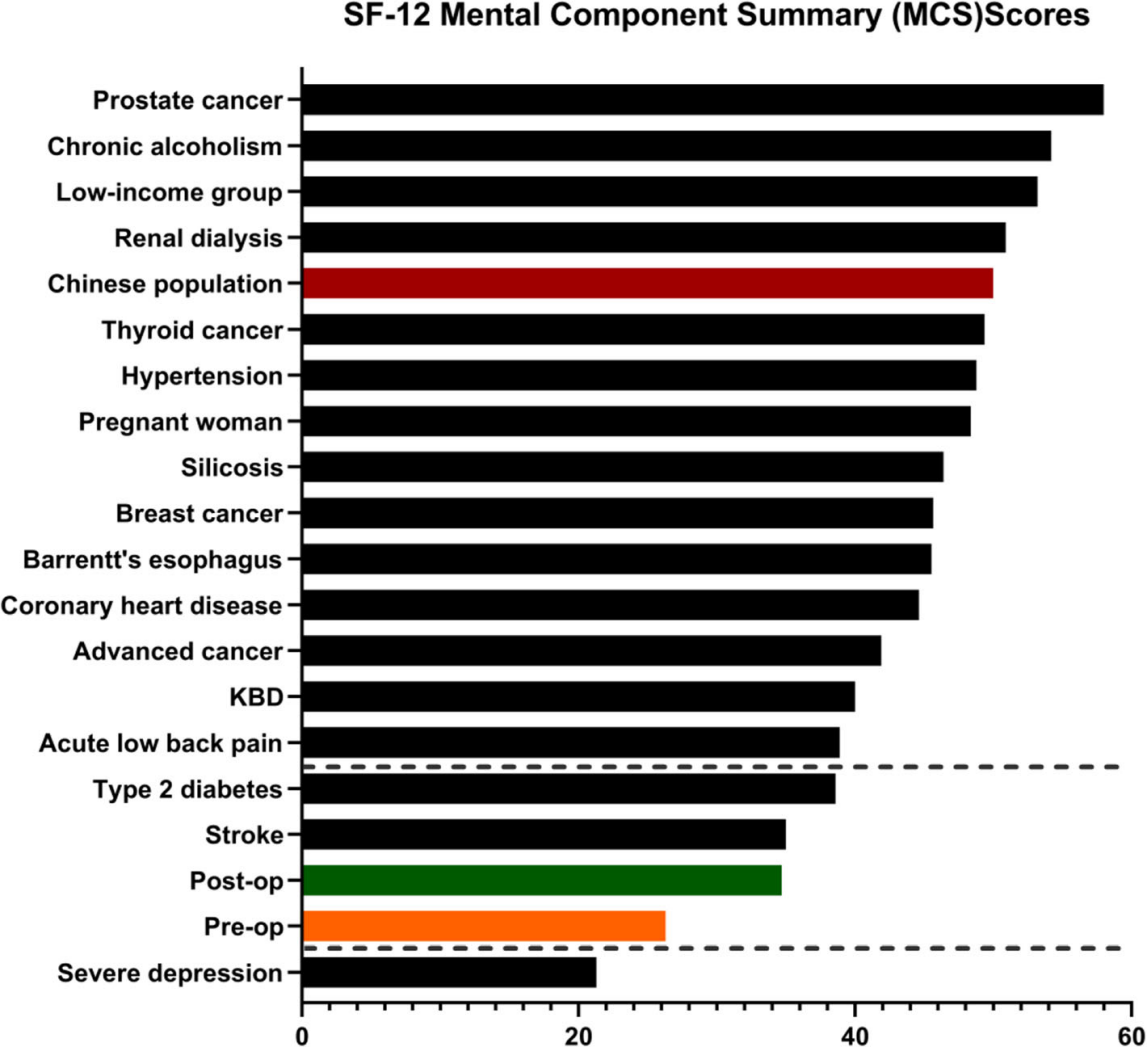
Mean SF-12 MCS scores according to diagnosis. Abbreviations: KBD, Kashin-Beck disease; COPD, Chronic Obstructive Pulmonary Disease. “Post-op” means that the fracture ends were healed smoothly. The medical condition above the upper dashed line or below the lower dashed line were associated with significantly (*p*<0.05) better or worse mental health, respectively, compared with pro-op and post-op MCS.

Humeral nonunion Patients have a low MCS score in all the literature reviewed (Fig. 3). Revision surgery does not significantly improve the mental health scores of patients with humeral nonunion, although the difference in scores before and after revision surgery were statistically significant (*p* < 0.001).The mean impact of humeral nonunion on mental health was comparable with the reported impact of stroke and type II diabetes (*p* > 0.05).Even more shocking is that the mean humeral nonunion MCS scores of pro-op and post-op were only superior to those of severe depression (all *p* < 0.001).

## Discussion

Nonunion is a chronic disease that can cause severe pain and disability in the limbs, which has a devastating effect on health-related QoL. To the best of our knowledge, this is the first study in which health-related QoL measures have been reported for patients with humeral nonunion.

Many clinical studies have shown that long bone nonunion causes severe pain and disability, which has major negative impact on patients’ daily life and QoL [4, 5, 10]. Femoral nonunion and tibial nonunion have a significantly higher attenuation effect on physical and psychological health than other orthopedic or non-orthopedic diseases, such as patellofemoral instability, shoulder instability, meniscus tear, ankle osteoarthritis, knee cartilage injury, rotator cuff tear, congestive heart failure, anterior cruciate ligament rupture, type II diabetes, myocardial infarction, depression and hypertension [5, 36]. Given that lower limbs long bone nonunion caused such a substantial burden, we investigated whether upper limb nonunion such as humeral nonunion causes similar low scores and has a profound negative effect on a patient’s health-related QoL.

Currently, the evaluation indexes of health-related QoL mainly include SF-36 scale, SF-12 scale, BPI scale, etc. The SF-12 is a brief version of SF-36 QoL scale, which is widely used in different countries and has a high correlation with the original health questionnaire SF-36. Lee et al. [37] verified that the Chinese version of SF-12 scale was also suitable for the Chinese population, which has the advantages of simple entry, concision, easy to understand and less operation time, therefore, it is often used to evaluate the QoL. The BPI scale has been widely used as a clinical assessment tool and translated into many languages. The Chinese version of BPI had been widely used in the assessment of acute and chronic pain, and cancer pain [38]. The Mayo elbow performance score (MEPS) was used to assess the elbow function of the patients in terms of range of activity, stability, pain and daily living ability.

The results indicated that after receiving humeral nonunion repair surgery, the patients’ PCS, MCS, BPI-S and BPI-I scores improved, with most significant changes in BPI-S and BPI-I. The higher score on the SF-12 PCS post-operation reflects good physical health, indicates no limitations in self-care and physical activities and satisfactory self-sensation in daily life. Notably, the impact on physical health was comparable with the reported impact of hypertension, even though the fracture was healing. The low score on the SF-12 MCS after revision operation reflects general mental health, indicates limitations in vitality as well as emotional and social function. During the follow-up, some patients no longer trusted doctors and their families after receiving multiple operations. Moreover, they had physical disability, anorexia, even suicidal thoughts, and some patients lost their job due to this. These findings were similar to those reported by Bhandari et al. and Loannou et al. that orthopedic trauma had substantial impact on perceived mental health and psychosocial function [39, 40]. Although the physical health consequences of humeral nonunion and other post-traumatic orthopedic conditions are perhaps more readily observable, the effects of such injuries on mental health may be under-recognized. Therefore, in the regular review of patients with humeral nonunion, the clinician should not only focus on the healing of the fractured callus but also screening and treatment to address psychological health. In this study, approximately 62.90 % of patients had local pain as the chief complaint, so the impact of pain on QoL may be the most important factor in follow-up visit. Successful revision surgery stabilizes the fracture and reduces the patient’s sensitivity to fracture pain, so the extent of pain’s interference on the QoL of the patient was significantly reduced in the current study.

Interestingly, the humeral nonunion score was found to be lower as compared to the SF-12 scale scores with other long bone nonunion before revision surgery [5, 6, 41]. The possible reasons for this phenomenon could be as follows. First, humeral nonunion due to failure of internal fixation often leads to severe appearance deformities or pseudo-articular formation, causing patients to fall into serious psychological obstacles [42]. Second, patients with nonunion of the lower extremities can travel by wheelchair and crutches, but there is no alternative for upper limb nonunion patients to perform some fine motor skills. Finally, in the era of electronic computerization when cell phone and computer use are indispensable (e.g., video games and online shopping), the humeral nonunion made the patient’s daily life inconvenient. Therefore, the critical impact on mentality and daily life is a potential contributing factor to their lower scores.

This study had several limitations: (i) This study only examined the final scores and functional outcomes for humeral nonunion, but the situation may be improved at a certain point in time. (ii) The number and geographical region of subjects were restively limited. (iii) Differences in cognition and comprehension of patients could have led to selection bias in final score. (iv) No data on patients with infected humeral nonunion were collected in this study, so the score of patients with humeral nonunion in this study was biased. (v) There is a lack of large population QoL survey in China, therefore we were unable to determine the extent of impact of humeral nonunion on the QoL of normal Chinese people.

## Conclusions

Successful revision surgery of humeral nonunion can greatly improve the subjective feeling and vitality of patients, but it cannot alleviate psychological trauma. Therefore, attention should be paid to the psychological needs of the humeral nonunion patients in rehabilitation treatment.

## Data Availability

The datasets generated and/or analyzed during the current study are not publicly available due to personal reasons, but are available from the corresponding author on reasonable request.

## Abbreviations

QoL: Wuality of life
MCS: Mental Component Summary
PCS: Physical Component Summary
BPI-S: Brief pain inventory-severity
BPI-I: Brief pain inventory- interference
MEPS: Mayo elbow performance score
ROM: Range of motion
SF-12: Short Form -12
FDA: US Food and Drug Administration
BMI: Body mass index
GH: General health
PF: Physiological function
RP: Physiological role
BP: Body pain
VT: Vitality
SF: Social function
RE: Emotional role
MH: Mental health
SD: Standard deviation
